# Outcome of a reproductive health advocacy mentoring intervention for staff of selected non- governmental organisations in Nigeria

**DOI:** 10.1186/s12913-015-0975-0

**Published:** 2015-08-11

**Authors:** Gloria. T. Momoh, Mojisola M. Oluwasanu, Olufemi L. Oduola, Grace E. Delano, Oladapo A. Ladipo

**Affiliations:** Association for Reproductive & Family Health, Qtr 815A, Army Officers Mess Road, Ikolaba, P.O. Box 30259 Secretariat Post Office, Ibadan, Oyo State Nigeria

**Keywords:** Advocacy, Non-governmental organisation, Reproductive health, Policy

## Abstract

**Background:**

Non-governmental organisations (NGOs) are expected to be in the vanguard, repositioning reproductive health as a central issue in population and development in Nigeria. However, most of them have insufficient knowledge or access to policy and planning processes necessary at engaging effectively with the government. This article highlights the processes and outcome of an intervention aimed at strengthening the capacity of 12 non-governmental organisations on advocacy and policy related activities with emphasis on reproductive health issues.

**Methods:**

The study employed a one group, pre and post test study design. Thirty six (36) staff from 12 NGOs was purposively selected and interviewed using a semi-structured questionnaire at baseline to assess their knowledge and level of involvement in reproductive health, advocacy and policy issues. In-depth interviews were conducted with 6 officials of the ministries of health and women affairs to document previous reproductive health and policy related collaborative efforts with the NGOs. Baseline findings were used in developing and implementing a capacity building intervention. A post intervention evaluation was conducted to assess the outcomes.

**Results:**

All respondents (100 %) had tertiary level education and were from a multidisciplinary background such as nursing (41.7 %) medicine (25 %) and administration (13.9 %). The mean knowledge score on advocacy and policy issues at pre-test and post test was 39.1 ± 17.6 and 76.2 ± 14.2 respectively (*p* = 0.00). Participants reported making use of advocacy methods and the three most utilized were Phone calls (28.1 %), Face to Face meetings (26 %) and networking with other organisations for stronger impact (17.1 %).

The outcome of their advocacy efforts include the provision of free air time by a television station to educate the populace on maternal health issues, donation of landed property to build a youth friendly centre, donation of a blog site for disseminating information on Reproductive health issues and training of other staff of their organisations on advocacy activities. The major challenges experienced by staff of the NGOs were financial (89 %) and time constraints (11 %).

**Conclusion:**

Empowered non-governmental organisations can effectively advocate for the implementation of reproductive health policies and programmes.

**Electronic supplementary material:**

The online version of this article (doi:10.1186/s12913-015-0975-0) contains supplementary material, which is available to authorized users.

## Background

The Reproductive Health (RH) situation in Nigeria is abysmal and the country has one of the worst bio-data in the world. It has a fast growing population, characterised by high infant, under five and maternal mortality ratios - 84/1000 live births, 163/1000 live births, and 576/100,000 live birth respectively [1, 2]. Ten percent of global maternal deaths occur in Nigeria although the country makes up only 1.7 % of the total world population. In other words, a Nigerian woman dies every 10 minutes from the complications of pregnancy [3]. Over one million Nigerian children will die before their fifth birthday, a figure that represents about 10 % of the global total [4] although many of these deaths are preventable.

The adolescent reproductive health situation is equally poor. The sexual and reproductive health needs of young persons are often unattended to and they have poor access to sexual reproductive health information and services. These result in risky sexual behaviours, teenage pregnancies, unsafe abortions, drug abuse and contraction of STIs including HIV/AIDS [5–7] According to the National Adolescent Reproductive Health Survey 2012, 20 % of male and 37 % of female adolescents within the age group of 15–19 years were already sexually active [7]. Early sexual initiation increases the risk of unintended pregnancies, complications and unsafe abortion. Eighty percent of all unintended pregnancies in Nigeria occur among young people and over 80 % of about 600,000 unsafe abortions are also among young people. Furthermore, complications from abortion account for 72% of all deaths in young women under age 19 [8], and half of all maternal deaths result from illegal abortions among Nigerian adolescents [8]. Forty four percent of new HIV infections have been recorded in this target group [9], with 80 % in adolescent females. This brings to fore the contribution of gender inequalities and reproductive right violation to the rate of contraction of HIV and poor Sexual Reproductive Health outcomes among this target group. Efforts to improve the reproductive health situation have been documented in several policies and programmes [10]. Following the country’s return to democratic rule, efforts have been deployed by the government to respect, protect and fulfil the reproductive rights of her citizens. Among such measures were the formulation of the National Reproductive Health Policy (2001) [11], National Family Life HIV&AIDS Education Program (2004) [12], National Gender Policy (2006) [13] and the Adolescent Health & Development Policy (2007)[14]. However; a wide gap still exists between policy intentions and programme implementation [15]. Enlisting meaningful political priority for safe motherhood and other RH issues in Nigeria is dependent on gaining the active support of state-and local-level political, social and religious leaders, as the federalized nature of the political system circumscribes the power of the national government [16]. Generating political priority for safe motherhood and other reproductive health issues in Nigeria has been a challenge as political leaders are burdened with thousands of issues to consider each year and have limited resources to deal with these problems [16]. Programming increasingly emphasizes the need for advocacy as a catalyst for mainstreaming and sustaining interest in sexuality and reproductive health programmes [17]. Advocacy is an action directed at changing the policies, positions or programs of any type of institution. It can also be defined as putting an issue on the agenda, providing a solution to the problem and building support to act on the problem and solution [17]. Advocacy is important in catalysing public opinion and support [18] and in turn this public support becomes central in the effective implementation of policies and programmes [19, 20].

Civil Society Organisations should be in the vanguard, positioning reproductive health as a central issue in population and development in Nigeria. They should be involved in policy dialogues to discuss development programmes but in reality, most of them have insufficient knowledge or access to policy and planning processes [21]. Some of these organisations lack the wherewithal to effectively implement developmental projects, negotiate and advocate effectively with the state governments and these has hampered their effectiveness. Non-governmental organisations in Nigeria are yet to come together as a cohesive and powerful agent of change, pushing the political and social systems to action [17]. The NGOs also lack a systematic framework for influencing the policy formulation processes [21].

Ogbogu et al*.* and Ritchie et al*.* in their publications highlighted the importance of strengthening the capacity of NGOs to influence relevant government policies and practice [21, 22]. In response to these gaps, Association for Reproductive and Family Health (ARFH) conducted an intervention targeting 12 NGOs in three Nigerian states. The intervention was designed to strengthen the capacity of these organisations to conduct advocacy and policy related activities focusing on Reproductive Health issues. This article describes the process and outcome of the intervention

## Methods

### Setting

The study was conducted in 3 Nigerian states. Kwara state is located in the north central geopolitical region of Nigeria and has a population of 2,371,089 [23]. Osun state is an inland state in south-western Nigeria and has a population of 3,423,535 while Ogun state is also in the South-western region of Nigeria with a population of 3,728,098 [23].

### Study design

The study employed a one group, pre and post test study design and data was obtained from selected staff of the NGOs before and after the intervention. Both qualitative (in-depth interview) and quantitative data collection methods (semi-structured questionnaire) were used.

Based on ARFH’s previous experience working with nongovernmental organisations (NGOs), a convenience sample of four NGOs/FBOs/professional organisations each were selected from the three states, totalling twelve partners. They were selected from a pool of NGOs/professional organisations who expressed need for their capacity to be built on the conduct of RH advocacy and policy related issues.

Trained research team members (GTM, MMO and OLO) conducted the interviews in the offices of the respondents using English as the language of communication. The interviews lasted approximately 40 minutes and these were tape recorded, transcribed and reviewed for accuracy. The findings of the baseline assessment guided the capacity building intervention for the NGOs.

### Baseline assessment

Baseline assessments of the 12 selected NGOs/FBOs/professional associations were conducted in the 3 project states. Instruments used for data collection at pre and post intervention (with modifications at the evaluation stage) were a 13-item open and closed-ended organisational capacity assessment tool (see Additional file 1) which documented the institution’s capability to implement the project and a semi structured questionnaire which consisted of 28 open and close-ended questions covering their demographic profiles, professional qualifications, training needs, the reproductive health advocacy and policy related programmes they had previously implemented, challenges encountered, and priority RH issues in the project states (see Additional file 2). In addition, an in-depth interview guide (see Additional file 3) was used to interview 6 key officials (2 per state) at the Ministries of Health and Women affairs to document previous collaborative efforts with the NGOs/ FBOs with emphasis on reproductive health advocacy and policy related issues. The questions focused on their awareness about the project, advocacy activities carried out by collaborating NGOs at the ministries, initial and current impression of ministries officials about the project, opinion about the project tenets and collaborative aspect of project strategy, lessons learned and project benefits. These tools were reviewed by peers and other professionals with skills in RH advocacy and policy related issues.

### The intervention

The intervention phase spanned 8 months comprising six key activities specifically a 5-day training programme and identification of key reproductive health needs in the states , mentoring, conduct of advocacy visits, formation and registration of state advocacy networks, monitoring and consultative meetings.

The 5-day training programme conducted for representatives of the selected NGOs lasted an average of 8 h daily. The activities were aimed at updating knowledge & strengthening the skills of trainees on advocacy issues. Thirty six participants attended the workshop. The capacity building programme focused on issues in reproductive health, steps in Advocacy process, policy issues, gender issues, courting the media, resource mobilization, networking, partnership and leadership issues. A main outcome of the training programmes was the ability of the participants to identify key RH issue in the project states using a Participatory Learning approach-“*The Pair Wise Ranking of Needs*”. The Pair wise ranking is a structured method for ranking a small list of items in priority order. It can help in prioritizing a small list as well as make decisions in a consensus-oriented manner [24]. To conduct the pair wise ranking, participants identified a maximum of 7 key reproductive health issues in their states using free listing. A pairwise matrix was constructed and each box in the matrix represented the intersection or pairing of two items. The team began the process by using consensus to determine which of the paired item had the most significant impact on the reproductive health status of populace using the following criteria *rate of occurrence, outcome of the reproductive health issue and the age groups affected. The process was repeated until the matrix was completed. The RH issue with the highest frequency was identified as the key RH issue of significance in the state.* Key reproductive health issues identified by each state are as outlined. *Ogun state*: Inclusion of Family Life HIV/AIDS Education (sexuality education) in curricula at all levels and the provision of Youth friendly services. *Kwara State*: Inclusion of Family Life HIV/AIDS Education (sexuality education) in curricula at all levels. Osun state: Reduction of maternal morbidity and mortality through the provision of Emergency Obstetric care at the primary and secondary health care facilities.

A 6-month work plan indicating the advocacy goal, objectives, activities, target audiences and timeline was developed in line with the key RH issues identified. This served as a guide for the conduct of subsequent advocacy activities in the project states. Two approaches were utilized in the conduct of the advocacy visits i.e. conduct of advocacy visits by the networks and joint advocacy visits by ARFH staff and the Networks. The target audiences for these advocacy activities were the legislators, policy makers, traditional, religious and opinion leaders, officials of reproductive health line ministries and the media.

In each of the project states, an advocacy network was formed. All the networks were registered with the State ministries of Women Affairs and Social Development as a criterion to functionality in their respective state, and the Ogun state advocacy network created a blog site to project its activities. The registration of the networks with the State ministries of Women Affairs and Social Development as well as the development of a blog site was a key outcome of the intervention.

Bi-monthly monitoring and consultative meetings were held with the networks to supervise the activities of mentees and also participate in their advocacy activities. Mentoring was a key capacity building activity on the project and this was aimed at strengthening the skills and competency of the participants to conduct advocacy programme. To accomplish the mentoring objectives, 4 strategic approaches were adopted specifically the participation of the NGO staff in a 2 day training and practical advocacy events to understudy the advocacy skills deployed by the facilitators, provision of resource materials, attachment to Mentors from ARFH coupled with ongoing mentoring through e-mails and telephone calls for six months. The mentees were expected to provide a progress update on a biweekly basis outlining key achievements and challenges experienced during the conduct of any advocacy event and Mentors were expected to provide technical assistance in addressing the challenges identified. During the six-month online mentoring programme, an average of 2 mails per mentee were received. Key factors which affected this approach were the limited internet connectivity in some of the states as well as the low skills of the participants in operating computers and internet facilities.

### Evaluation of outcome

Final evaluation was conducted 6 months after the intervention. Semi-structured questionnaires were administered to 30 trained staff of the partner NGOs. Compared to the number interviewed at the baseline assessment (36), this number was lower due to the inability of the interviewers to contact some of the trained staff at final evaluation. Utilizing an in-depth interview guide, the opinions of 6 representatives of State Ministries of Health and Women Affairs were also sought regarding project outcome.

### Data analysis

The data obtained from the semi-structured questionnaires were coded and analyzed using SPSS version 15 software to generate descriptive statistics specifically frequencies. Using a paired sample t-test at 5 % level of significance and 95 % confidence interval, we compared the mean knowledge score of the trainees before and after the training. The in-depth interview discussions were manually transcribed and summarized thematically.

### Ethics

Ethics Review Committee of the Association for Reproductive and Family Health, Ibadan, Nigeria (http://arfh-ng.org/) reviewed and approved the proposal. Participation was voluntary with confidentiality assured. The non- governmental organisations and the respondents received detailed information on the objective of the study. Verbal consent was obtained from respondents before questionnaires were administered or interviews conducted. Individual identifiers such as names were not included in the data collection instrument.

## Results

### Demographic characteristics of respondents

A total of 36 and 30 trained staff were interviewed at baseline and final evaluation respectively. All the participants had tertiary level education and were from a multidisciplinary background such as medicine, nursing, medical sciences, social sciences, administration etc. There were more males than females (55.6 % and 60 % males at baseline and final evaluation respectively). More than two-thirds of them were aged 40 and above, Table 1 gives a detail of the demographic characteristics.

**Table 1:** Demographic characteristics of respondents

	Baseline assessment	Final evaluation
Socio-demographic characteristics	No (%)	No (%)
Sex		
Male	20 (55.6)	18 (60)
Female	16 (44.4)	12 (40)
Age		
20–29	4 (11.1)	2 (6.7)
30–39	7 (19.4)	7
40–49	10 (27.7)	10
50 and above	15 (41.8)	11
Religion		
Christianity	29 (80.6)	23 (76.7)
Islam	7 (19.4)	7 (23.3)
Marital status		
Married	28 (77.8)	24 (80.0)
Single	4 (11.0)	2 (6.7)
Separated	2 (5.6)	2 (6.7)
Widowed	2 (5.6)	2 (6.7)
Academic Background	5 (13.9)	4 (13.3)
Administration	1 (2.8)	1 (3.3)
Medical Sciences	2 (5.6)	2 (6.7)
Arts	3 (8.3)	2 (6.7)
Social Sciences	9 (25)	7 (23.3)
Medicine	15 (41.7)	13 (43.3 )
Nursing	1 (2.8)	1 (3.3)
Education		

### Organisational assessment on reproductive health advocacy and policy issues

The findings of the organisational assessment revealed that none of the NGOs had implemented projects focusing solely on RH advocacy and policy related issues. In addition, none of the proposed participants had participated in training programmes on advocacy or policy related issues though all of them had utilized some advocacy strategies/methodologies in RH programming. The findings of the in-depth interviews at baseline revealed that, the ministries had partnered with 5 out of the 12 organisations in implementing RH project.

#### Knowledge of trained staff about advocacy-related issues before and after training

The final evaluation, the knowledge of the trained NGOs staff had been enhanced in several ways. The pre assessment on knowledge of advocacy and policy issues before the training revealed a minimum and maximum score of 10 and 73 respectively, mean was 39.1 ± 17.6. At post test, the minimum and maximum score was 44 and 99 respectively and mean was 76.2 ± 14.2. Further analysis revealed that the difference in the mean scores at pre and post test was statistically significant t (35) = −12.65, *p* = 0.00 (two-tailed).

Table 2 shows the proportion of trained staff reporting improved knowledge on advocacy-related issues before and after training. Almost all of the trained staff (96.7 %) of participating organisations reported that they had conducted step-down training for other members of staff in their work places and for most of them (86.2 %); the focus of the training was advocacy and sensitization.

**Table 2:** Proportion of trained staff reporting improved knowledge on advocacy-related issues before and after training

S/N	Advocacy related issues	Percentage at baseline	Percentage after training	Percentage that found the topics useful
1	Advocacy processes	34.3	100	96.7
2	Advocacy Methodology	25.7	96.7	96.6
2	Community mobilization and participation in reproductive sexual health/family planning programmes	-	96.7	93.3
3	Continuity and sustainability plan	25.7	90	93.3
4	Behaviour Change Communication	37.1	96.7	93.4
5	Mentoring	20.0	96.7	93.4
6	Networking for impact	34.3	100	93.3
7	Team building	37.1	96.7	93.3
8	Decision making	40.0	93.3	93.3
9	Gender analysis	42.9	90	83.3
10	Gender and development Concepts	42.9	86.7	83.3
11	Fund raising	-	96.7	96.7
12	Communication strategies for RH/FP advocacy	-	96.7	93.3
13	Management system	-	96.7	93.3
14	Conflict management	-	100	96.6
15	Monitoring and evaluation	-	100	96.7
16	Report writing	-	100	93.4

#### Advocacy methodologies utilized by the trained personnel

Majority (93.3 %) of those trained conducted advocacy activities after the training. These activities included visits to media houses (93.1 %), sensitization of health workers (62.1 %), advocacy to stakeholders (37.9 %), advocacy to the State Ministry of Women Affairs (17.2 %) and health talks (6.8 %). The targets of the advocacy activities included the media (71.4 %), health workers (78.6 %), church members (46.3 %), State Ministry officials (42.9 %), community leaders (35.7 %), market women (10.7 %) and Local Government Chairmen (10.7 %). Table 3 gives a detail of advocacy activities conducted by the trained staff. Majority (86.7 %) of those trained indicated that there have been changes in the way they conduct advocacy activities. When asked to specify the changes, about half of them (48 %) mentioned that they had started to use the mass media while 6.7 % mentioned the use of lobbying and phone calls to convey advocacy messages. The Management Information System (MIS) forms designed to monitor their activities were analyzed to estimate the frequency of use of each advocacy method. Table 3 gives the detail of the methods used and frequency of use.

**Table 3:** Advocacy methods utilized by trained personnel

Advocacy method	State						Total	
	Ogun		Osun		Kwara			
	No	%	No	%	No	%	No	%
Phone calls	27	27.3	53	26.6	12	41.4	92	28.1
Face to face visits	25	25.3	51	25.6	9	31.0	85	26.0
Lobbying	20	20.2	6	3.0			26	8.0
Provision of complimentary materials	2	2.0	30	15.1	2	6.9	34	10.4
Press work/media coverage	2	2.0	-	-	-	-	2	0.6
Campaign/rally	1	1.0			2	6.9	3	0.9
Negotiation	1	1.0	18	9.1			19	5.8
Consensus building	2	2.0	5	2.5	3	10.3	10	3.1
Networking	19	19.2	36	18.1	1	3.5	56	17.1
Total	99		199		29		327	

#### Reported outcomes of the advocacy activities

The advocacy activities got a lot of positive responses from the target population. Close to half (46.6 %) of the trained personnel said the activities were very successful and encouraging, while a third added that it attracted mutual willingness and support and a tenth of them also reported increased awareness and support from media houses. Some of the outcomes of the intervention were the granting of free air time by the media to discuss issues on Reproductive health with the public, provision of a landed property for the construction of a youth friendly centre and financial commitment of resources towards the actualisation of the identified reproductive health concerns of the states.

Furthermore, the project facilitated better collaboration and networking among the 12 organisations whose capacities was built. In each state, they formed Reproductive health advocacy networks which they registered with the State Ministries of Women Affairs & Social Development. These advocacy networks developed a 6 month action plan to address pertinent reproductive health issues in their respective states, and members of the networks contributed their personal resources to implement some of the activities on the work plans. As a result of this collaboration, several benefits have accrued to each participating organisation, which include greater recognition by the government and donor agencies, use of the mass media at reduced prices and creation of a forum for sharing ideas and materials, which inevitably led to increased knowledge of participating individuals and links to existing opportunities.

In-depth interviews conducted with the personnel in the ministries of Health and Women Affairs corroborated the reported findings by the CSO advocacy networks. All the ministry officials interviewed affirmed that they were aware of the project and expressed positive opinions about the activities of the advocacy networks. For instance a government official in Ogun, stated that the project has been *“Very impressive….”*

The most prominent outcome of the project was its ability to facilitate linkage and partnership between the participating CSOs and these Ministries. According to a government official in Osun State ‘*the collaborating NGOs and CBOs have been able to work together to develop programme implementation objectives*’. In addition, a government official in Kwara state mentioned that networks have been able to *identify health problems, plan advocacy visits and propose solutions to policy makers*.

The Ministry Officials stated that they learnt some lessons on the project. According to a government official at the Ministry of Women Affairs and Social Welfare in Kwara state ‘*the project demonstrated the advantage of collaboration and networking*. Kwara state Ministry of Health officials opined that ‘*the project helped in identifying the most important health problems in the state’*. Table 4 shows details of advocacy activities conducted by trained staff as reported by ministry officials.

**Table 4:** Advocacy activities conducted in each state as reported by the ministry officials

State	Designation of ministry official interviewed	Advocacy activities carried out by the CSO advocacy network	Number of such events that have direct link with policy making	Roles played by Ministry Officials in Linking Advocacy Event to Policy Making
Ogun	Deputy Director of Women Affairs and Social Welfare, State Ministry of Health, Abeokuta South	Phone calls	ALL	Collaborating and facilitating the meeting of the NGOs
		Courtesy visit		
		Lobbying		
		Supply of complimentary materials		
		Campaigns		
		Negotiations		
		Consensus building		
		Networking		
Osun	Director PHC, State Ministry of Health	Phone calls	ALL	Direct introduction to policy makers
		Courtesy visit		
		Campaigns		
Kwara	1) FP Coordinator, State Ministry of Health	Phone calls	NIL	Attempting to meet the Commissioner for health but political atmosphere was not been conducive
		Courtesy visit		
	2) Chief Public Health Nursing Officer, State Ministry of Health	Lobbying		
		Supply of complimentary materials		
		Campaign		
		Negotiations		
		Consensus building		
		Networking		

#### Reported challenges faced in the conduct of advocacy activities

More than three-fifths (63 %) of the trained staff reported that they had experienced challenges during the advocacy process. The major challenges faced were financial (89 %) and time constraints (11 %). According to a respondent from Ogun state, ‘*funding every step was becoming more difficult*’. Another respondent from Osun state stated that ‘*Most policy makers were not readily available resulting in the waste of time and resources*’. Financial challenges were also experienced by Officials of the line Ministries. This reflected in responses such as ‘*lack of vehicles for transportation’* and *‘inadequate personnel’*

Financial challenges were handled mainly by making voluntary donations among network members (64.7 %) and soliciting for support from media houses (29.4 %). Other challenges were handled through perseverance (13.3 %) and lobbying to ensure that visits made were not futile. A respondent also mentioned that team members made personal commitment in terms of time in order to overcome the constraints.

Another challenge experienced on the project was the coincidence of the project year with the electoral year. Most of the target audiences identified were policymakers who were newly appointed and needed time to get acquainted with the activities in their ministries or local government councils. In addition, some policy makers in the transition committees at the local government or state government had expressed support for the project initiatives but were replaced and this necessitate the conduct of several repeat advocacy visits.

## Discussion

Our findings indicate that building the capacity of NGO staff in Reproductive health advocacy can lead to a change in the Reproductive health policy and programme service delivery environment. The finding from this study is corroborated by data from the study by Amadi et al*.* [17] where church leaders had their capacity enhanced and they effectively advocated to duty bearers on necessary changes within their communities. The study by Amadi et al. [17], also found that advocacy programming is successful when it is built on community/grass root experiences using a participatory process that involves diverse stakeholders.

The need to strengthen the capacity of non-governmental organisations to advocate for implementation of policies and programmes cannot be overemphasized. This is important for the effectiveness of advocates in achieving the identified advocacy goal. Capacity building activities targeting non-governmental organisations should emphasise training and institutional building in line with identified advocacy actions [25]. Networking i.e. initiating and maintaining contact with other organisation with shared goals it also a key factor requisite for a successful advocacy initiative [26, 27].

Mentoring has been a viable method for fostering early professional growth among students [28]. It has been transformed from traditional master-apprentice relationships into multiple developmental relationships that extend beyond functional, organisational, and geographic boundaries. The Internet provides one mechanism for protégés to identify mentors to help navigate career opportunities. The mentoring experience can provide access to role models, individual open discussion, and reliable resources for practical information [28]. This innovative approach was effectively utilized on this intervention to enhance the advocacy skills of the trainees. Capacity building activities targeting professionals in developing countries should explore internet mentoring as a key strategy for skills and knowledge acquisition and transfer.

Leadership is a very key factor for a successful advocacy effort [21]. The team in Kwara state reported the lowest number of advocacy events conducted as well as the outcomes achieved. This may not be unconnected to the leadership and its inability to effectively mobilize other members of the team in achieving the advocacy targets.

Challenges experienced by the trainees in conducting advocacy activities were financial and time constraints. This is in line with other research findings [23]. Other challenges include the sustainability of advocacy initiatives and networks after the expiration of donor funding. After this intervention study, ARFH was awarded a grant by the Future Institute, USA and some of the partner organisations received continued support for their advocacy activities from this grant. A more sustainable strategy in addressing this challenge is to identify other options for funding such as membership levies, donations and grants from public and private institutions.

## Conclusion

The project created a platform for interaction between the people at the community level with policy makers and traditional leaders on RH issues that have been identified by fellow community members with each group reaching a consensus that these required repositioning. This has created an enabling environment that will foster policy changes in the project states.

In addition, it has strengthened the capacity of the non-governmental organisations to conduct advocacy activities which provide supportive environment for the implementation of RH programmes in the project states. It is hoped that this would lead to the development of other innovative advocacy programmes that will act as catalyst for political and financial support for Reproductive health issues in Nigeria.

The concept of advocacy to policymakers and traditional leaders is a prerequisite for transforming policy initiation to programs that will impact on the sexual reproductive health of the citizenry.

This study has a number of limitations such as the lack of comparison group, self reported nature of the survey, and the short duration of the intervention period. To address this limitation, the findings from the NGOs were triangulated with data from the in-depth interviews with the officials of the ministries and policy makers. The Association for Reproductive and Family Health is committed to providing further support towards strengthening NGOs capability for a continuous engagement with duty bearers.

## Additional files

Additional file 1:
**NGO Capacity Assessment, docx, Organisational Capacity assessment tool f for NGOs.** (DOC 74 kb) Additional file 2:
**Semi-structured questionnaire, doc, Semi-structured questionnaire for NGO staff.** (DOC 96 kb) Additional file 3:
**Indepth interview guide, doc, Indepth interview guide for ministry official.** (DOC 61 kb)

## Abbreviations

AIDS: Acquired immune deficiency syndrome
ARFH: Association for reproductive and family health
FBO: Faith based organisation
HIV: Human immunodeficiency virus
MIS: Management information system
NARHS: National adolescent reproductive health survey
NGO: Non-governmental organisations
RH: Reproductive health
STI: Sexually transmitted infections
