# Supplementary material for: Outcome of a reproductive health advocacy mentoring intervention for staff of selected non- governmental organisations in Nigeria
Source: BMC Health Serv Res. 2015 Aug 11;15:314. doi: 10.1186/s12913-015-0975-0 (PMC4560878; doi:10.1186/s12913-015-0975-0)
Supplement: Additional file 1: — NGO Capacity Assessment, docx, Organisational Capacity assessment tool f for NGOs. (DOC 74 kb) [file 12913_2015_975_MOESM1_ESM.doc]

**ASSOCIATION FOR REPRODUCTIVE AND FAMILY HEALTH (ARFH)**

**IKOLABA G.R.A., IBADAN**

**Capacity Building of CSO Partners to Carry out Reproductive Health /Family Planning Advocacy and Policy Related Activities**

#### Organizational Capacity Assessment Tool For NGOs/CSOs/FBOs

##### Introduction/Instruction.

This survey is being conducted by the **Association for Reproductive and Family Health (ARFH), Ikolaba, Ibadan in collaboration with partner NGOs/CBOs/FBOs in Kwara, Ogun and Osun States.** ARFH has a vision of enhanced sexual and reproductive health and right of individuals and couples in Nigeria and else where in Africa.

The project titled **“Capacity Building of CSO Partners to Carry out Reproductive Health /Family Planning Advocacy and Policy Related Activities”** is aimed at creating an enabling environment in which effective and efficient RH/FP programmes can function and it will involve capacity building of staff of NGO/CSOs/FBOs to conduct advocacy and policy related activities.

We will like to seek your opinion regarding those issues that have to do with advocating for policies on Population and Reproductive health/family planning. In order to effectively involve the relevant existing structures from the onset and to obtain information that will aid planning and implementation of the project, we will appreciate if you can assist in completing this guide

There are no right or wrong responses and be assured that anything written will be treated with strict confidentiality and used only for the purpose of research, planning and implementation of the project. Thank you in advance for your time and input.

# **PROJECT IDENTIFICATION**

State: __________________________________________________________________________

LGA: __________________________________________________________________________

Sector: 1. Urban [ ] 2. Semi-urban [ ] 3. Rural [ ]

Name and Address of CBO/NGO/FBO : _____________________________________________

_______________________________________________________________________________

_______________________________________________________________________________

_______________________________________________________________________________

_______________________________________________________________________________

## Designation of person being interviewed: ____________________________________________

Field Supervisor’s Name: ____________________________Signature_________Date_________

# **This project is being supported by Enabling HIV/AIDS+TB and Social Sector Environment (ENHANSE)**

# **SECTION A: BASIC INFORMATION**

Name of Organization**:_______________________________________________________**

_________________________________________________________________________

Name of Contact Person :____________________________________________________

Address: _________________________________________________________________

Phone/fax: ____________________________E-mail: ______________________________

Mission of Organization: ____________________________________________________

Staff Strength: ____________________________________________________________

No of staff on pay roll: _____________________________________________________

No of staff volunteering/part time: ____________________________________________

Existing Structure in the Organization (Organogram)

### SECTION B. INFORMATION NEEDS ASSESSMENT (responses can be written on additional sheets of paper if space provided is not adequate)

1. Could you please describe the focus of organization: E.g. HIV/AIDS; RH /FP, Youth, Women Empowerment; Research etc
2. If the organization has been involved in Reproductive Health (RH) activities, could you please list RH projects implemented within the last four (4) years with dates, general objectives and funding agencies:
3. State types of community involvement:
4. Please state major reproductive health issues you think are peculiar in this state?

5a. Is your organization currently involved in the implementation of advocacy and policy

related activities in the area of Reproductive health and family planning?

5b. If yes, does there exist any structure for networking with other institutions or

organizations that have organized any advocacy event that resulted in a major policy or

operational changes?

5c. If Yes, list these organizations and their roles:

5d. What are the benefits accruing to the organization by reason of the networking?

5e. If there is no structure for networking in existence, why?

6a. How would you rate your organization in terms of implementation of advocacy and

policy related activities in the area of Reproductive health and family planning, using a

rating scale of 1 – 5 (1 lowest, 5 highest)

| ACTIVITIES | RATING SCALE | | | | |
| --- | --- | --- | --- | --- | --- |
|  | 1 | 2 | 3 | 4 | 5 |
| Advocacy |  |  |  |  |  |
| Policy related activities |  |  |  |  |  |
| Trainings |  |  |  |  |  |
| Others (specify) |  |  |  |  |  |

6b. What are your areas of strengths?

6c. What are your areas of weaknesses?

6d. State challenges faced in implementing advocacy:

6e. State challenges faced in implementing policy related activities:

1. What major RH/FP changes would you want to make?

8. What major RH/FP policy (ies) would you wish to be in place?

7. Type of advocacy activities carried out in the organization:

| S/N | Types of advocacy activities | Tick the appropriate answer: |
| --- | --- | --- |
| 1 | Phone calls |  |
| 2 | Courtesy Visits |  |
| 3. | Lobbying |  |
| 4. | Provision of complimentary materials |  |
| 5. | Campaign |  |
| 6. | Negotiations |  |
| 7. | Consensus building |  |
| 8. | Networking |  |
| 9. | Others (specify ___________________________) |  |
| 10. | Others (specify____________________________) |  |

8a. Does your organization have an established office with office equipment?

8b. If yes, how many rooms?

8c. Does your organization have adequate staff?

8d. If yes, how many are they?

8e. Tick type of office equipment available:

| **S/N** | **Equipment** | **Available** | **Not available** |
| --- | --- | --- | --- |
| 1 | Office furniture |  |  |
| 2. | Separate reception area |  |  |
| 3. | Separate Office for staff |  |  |
| 4. | Filing cabinet (s) |  |  |
| 5. | Office shelves |  |  |
| 5. | Computers |  |  |
| 6. | Printers |  |  |
| 7. | Internet services |  |  |
| 8. | Flip chart stand |  |  |
| 9. | Projector (specify type) _______________ |  |  |
| 10. | Video/ VCD machine |  |  |
| 11. | Electricity generating set |  |  |
| 12. | Television |  |  |
| 13. | Tape recorder(s) |  |  |
| 14. | Public address system / megaphone |  |  |
| 15 | Steel camera |  |  |
| 16 | Stabilizer |  |  |
| 17. | UPS |  |  |
| 18. | Others (specify_______________________) |  |  |

9. Does your organization prepare annual budgets?

1. Does your organization possess the ability to mobilize funds?

11. Does your organization have a strategic plan?

12a. Has your organization ever been involved in scaling up reproductive health /family

planning advocacy and policy related activities?

12b. If yes, state when:

12b. Could you please list the strategies adopted:

13a. How often does your organization carry out reproductive health/family planning?

advocacy activities?

13b. What has been the outcome of your activities?

**Thank you for your time and input**
