# Supplementary material for: Outcome of a reproductive health advocacy mentoring intervention for staff of selected non- governmental organisations in Nigeria
Source: BMC Health Serv Res. 2015 Aug 11;15:314. doi: 10.1186/s12913-015-0975-0 (PMC4560878; doi:10.1186/s12913-015-0975-0)
Supplement: Additional file 2: — Semi-structured questionnaire, doc, Semi-structured questionnaire for NGO staff. (DOC 96 kb) [file 12913_2015_975_MOESM2_ESM.doc]

ASSOCIATION FOR REPRODUCTIVE AND FAMILY HEALTH (ARFH)

**IKOLABA G.R.A., IBADAN**

Capacity Building of CSO Partners to Carry out Reproductive Health /Family Planning Advocacy and Policy Related Activities

QUESTIONNAIRE FOR STAFF OF NGOs/CSOs/FBOs

##### Introduction

Greetings, my name is ______________________________________. The Association for Reproductive and Family Health (ARFH), Ikolaba Ibadan implemented a project

titled “Capacity Building of CSO Partners to Carry out Reproductive Health /Family Planning Advocacy and Policy Related Activities” in collaboration with some NGOs/CSOs/FBOs in Kwara, Ogun and Osun states. This project was designed to create an enabling environment for effective and efficient RH/FP programmes by building the capacity of staff of NGOs/CSOs/FBOs to conduct advocacy and policy related activities.

In order for ARFH to effectively assess the outcome of the program, we request that you respond to the following questions factually and honestly.

# This project is being supported by Enabling HIV/AIDS+TB and Social Sector Environment

# (ENHANSE)

Thank you

###### Section A: Demographic Information

1. Sex 1. Male 2. Female

2. Age: 1. Below 20 years 2. 20-24 3. 25 - 29

4. 30 – 34 5. 35- 39 6. 40-49 7. 50 and above

3. Religion: 1. Islam 2. Christianity (specify denomination)________

3. Traditional religion 4. Others (specify____________

4. Marital Status:

1. Single / Never Married

2. Married

3. Separated

4. Divorced

5. Widowed

5. Highest educational qualification / Year:_________________________________

6. What are your main job descriptions / responsibilities (please tick all that apply)

1. Programme implementation

2. Managerial/ Administration

3. Supervisory

4. Research

5. Training

6. Others (specify) ____________________________________________

### Section C: Training Experience

7. Have you attended any post qualification-training program related to reproductive health and family planning advocacy and policy related activities in the last nine months? 1. Yes 2. No

8. If yes to Question 7, list any training/programs you attended related to Reproductive Health and family planning advocacy and policy related activities **in the last nine months.**

### Reproductive Health, Family Planning and advocacy training Programmes attended (Pls list the training programmes attended in relation to reproductive health, family planning and advocacy in the last nine months):

| S/N | **Year** | **Course Title** | **Duration** | **Organized by** |
| --- | --- | --- | --- | --- |
|  |  |  |  |  |
|  |  |  |  |  |
|  |  |  |  |  |
|  |  |  |  |  |
|  |  |  |  |  |
|  |  |  |  |  |
|  |  |  |  |  |
|  |  |  |  |  |

9. Please indicate your training experience in the following Reproductive health and Family Planning issues

| **S/N** | **Reproductive and Family planning issues** | Did you receive any training on any of these in the last nine months | | How useful was the training to your practice | | |
| --- | --- | --- | --- | --- | --- | --- |
|  |  | Yes | No | Very useful | Useful | Not useful |
| i | Current trend and advancement in family planning |  |  |  |  |  |
| ii | Current trend and advancement in reproductive health |  |  |  |  |  |
| iii | Adolescents reproductive sexual health |  |  |  |  |  |
| iv | Safe motherhood |  |  |  |  |  |
| V | Post Abortion care |  |  |  |  |  |
| vi | Male involvement in reproductive health |  |  |  |  |  |
| vii | Contraceptive update |  |  |  |  |  |
| viii | Population issues |  |  |  |  |  |
| ix | Inter personal communication |  |  |  |  |  |
| x | Social and peer support |  |  |  |  |  |
| xi | Gender issues |  |  |  |  |  |
| xii | Counseling |  |  |  |  |  |
| xiii | Behaviour Change communication |  |  |  |  |  |
| xiv | Demand creation |  |  |  |  |  |

Specifically in relation to your training experience on Reproductive health and Family Planning issues (as indicated above):

10. How was the knowledge gained utilized? *_______________________________*

*___________________________________________________________________________*

11. Has there been any change in the way you carry out your reproductive health and

family planning activities ?

1. Yes 2. No

12. If yes please specify______________________________________________

13 Did you conduct any step down training in your Organization? Yes ( ) No ( )

(i). If yes, what was the focus of the step down training? ………………………………

…………………………………………………………………………………………….

(ii). If No, Why? .......................................................................................................…..

…………………………………………………………………………………..…………

…………………………………………………………………………………………….

### Advocacy and Policy Related issues

14. Please indicate your training experience in the following Advocacy and policy related activities

| **S/N** | **ADVOCACY** | Did you receive any training on any of these in the last nine months | | How useful was the training to your practice | | |
| --- | --- | --- | --- | --- | --- | --- |
|  |  | Yes | No | Very useful | Useful | Not useful |
| i | Advocacy Processes |  |  |  |  |  |
| ii | Advocacy methodology |  |  |  |  |  |
| iii | Resource mobilization in reproductive sexual health / family planning programmes |  |  |  |  |  |
| iv | Continuity and sustainability plan |  |  |  |  |  |
| v | Behaviour Change Communication |  |  |  |  |  |
| vi | Mentoring |  |  |  |  |  |
| vii | Networking for impact |  |  |  |  |  |
| viii | Team building |  |  |  |  |  |
| ix | Decision making |  |  |  |  |  |
| x | Gender analysis |  |  |  |  |  |
| xi | Gender and development Concepts |  |  |  |  |  |

Specifically in relation to your training experience on Advocacy and policy related activities:

15. How was the knowledge gained utilized? _________________________________

16 (i). Did you conduct any step down training in your Organization? Yes ( ) No ( )

(ii). If yes, what was the focus of the step down training?______________________

______________________________________________________________________

17. If No, Why?________________________________________________________

______________________________________________________________________

______________________________________________________________________

18(i). Have you been involved in any advocacy activity after the training?

1. Yes 2. No

(ii) If yes how many______________________________________________

(iii) Specify the type of advocacy activity (ies)

a._______________________________________________

b._______________________________________________

c.______________________________________________

d. ______________________________________________

19. Who was/were the target(s) of the advocacy?

a. ________________________________________________

b. _______________________________________________

c. _______________________________________________

d._______________________________________________

e. _______________________________________________

20. What were the steps involved in the advocacy?

a. ____________________________________________

b. ____________________________________________

c ____________________________________________

d____________________________________________

e. ____________________________________________

21. What was the outcome of the advocacy? ________________________________

________________________________________________________________

_________________________________________________________________

_________________________________________________________________

22. (i). Did you face any challenge in the process of advocacy?

1. Yes 2. No (If No, go to Ques 24)

(ii) If yes, what was the challenge? __________________________________

__________________________________________________________________

__________________________________________________________________

(iii) How did you handle it?

1. _______________________________________________
2. _______________________________________________
3. _______________________________________________

23. Do you have any plans for advocacy activities in future?

1. Yes 2. No (If No, go to Q 28)

24. If yes, please describe those activities.

a. _______________________________________________

1. _______________________________________________
2. _______________________________________________

25. How do you plan to go about the advocacy activities? (Please give a brief description) ________________________________________________________________________

**________________________________________________________________________**

**_______________________________________________________________________________________________**

**________________________________________________________________________________________________**

### Sections D: Recommendations for Project sustainability

26. What measure would you suggest should be put in place to ensure the sustainability of the current initiative / How do you think this project can be sustained?

________________________________________________________________________________________________________________________________________________

________________________________________________________________________

________________________________________________________________________

27. How do you think you can contribute towards the sustainability of the project?

## 28. Any other comment:

________________________________________________________________________

________________________________________________________________________

## _____________________________________________________________

## Thank you for your response
