# Supplementary material for: Outcome of a reproductive health advocacy mentoring intervention for staff of selected non- governmental organisations in Nigeria
Source: BMC Health Serv Res. 2015 Aug 11;15:314. doi: 10.1186/s12913-015-0975-0 (PMC4560878; doi:10.1186/s12913-015-0975-0)
Supplement: Additional file 3: — Indepth interview guide, doc, Indepth interview guide for ministry official. (DOC 61 kb) [file 12913_2015_975_MOESM3_ESM.doc]

ASSOCIATION FOR REPRODUCTIVE AND FAMILY HEALTH, IKOLABA, IBADAN, OYO STATE

**Capacity Building of CSO Partners to Carry out Reproductive Health / Family Planning Advocacy and Policy Related Activities**

**IN DEPTH INTERVIEW WITH PERMANENT SECRETARY/DIRECTOR PHC, STATE MINISTRIES OF HEALTH**

**AND WOMEN AFFAIRS**

##### Introduction /Instruction.

This interview is being administered by the **Association for Reproductive and Family Health (ARFH), Ikolaba, Ibadan,** Oyo State Nigeria. ARFH has a vision of enhanced sexual and reproductive health and right of individuals and couples in Nigeria and else where in Africa.

ARFH implemented a project titled “**Capacity Building of CSO Partners to Carry out Reproductive Health /Family Planning Advocacy and Policy Related Activities**” in collaboration with some NGOs/CSOs/FBOs in Kwara, Ogun and Osun states. This project was designed to create an enabling environment for effective and efficient RH/FP programmes by building the capacity of staff of NGOs/CSOs/FBOs to conduct advocacy and policy related activities.

This interview is designed to evaluate the outcome of the project on the NGOs, the community and the ministry. The information obtained from you will help us to effectively evaluate the project and will also be used planning and implementation of future projects. We will therefore appreciate if you can assist in completing this guide

Thank You Sir/Madam in advance for your time and input.

# PROJECT IDENTIFICATION

State: ____________________________________________________________

LGA: _____________________________________________________________

Sector: 1. Urban [ ] 2. Semi-urban [ ] 3. Rural [ ]

Designation of Interviewee: _________________________________________

Address of Interviewee: ____________________________________________

# Name of Field Supervisor: ____________________Signature: _________Date______

# This project is being supported by Enabling HIV/AIDS+TB and Social Sector Environment (ENHANSE)

1a. As a key officer in the Ministry of Health / Ministry of Women Affairs and a key stakeholder in the health sector of the state, are you aware of any collaboration between Association for Reproductive and Family Health and some CSOs, CBOs and FBOs on the project, “Capacity Building of CSO Partners to Carry out Reproductive Health /Family Planning Advocacy and Policy Related Activities” – ENHANSE project

1. Yes 2. No

2a. If yes how did you learn of this collaboration?

b. What was your first impression about the ENHANSE project?

___________________________________________________________________________

c. What is your impression of the project now- nine months after project implementation?

___________________________________________________________________________

3a. In what area(s) has your Ministry been involved in the project implementation?

___________________________________________________________________________

b. What is your opinion about the level of involvement of your Ministry in the project? ___________________________________________________________________________

4a. What is the opinion of your Ministry about the tenets of the project particularly

the capacity building of NGOs/CBOs/FBOs in advocacy activities?

___________________________________________________________________________

b. What is the opinion of the Ministry about the tenets of the project particularly the capacity building of NGOs/CBOs/FBOs in policy related activities?

___________________________________________________________________________

5a. What are your views about the collaborative aspect of the project strategy? Did it

work well? ______________________________________________________________

___________________________________________________________________________

1. If it worked well, state those areas that worked well:

___________________________________________________________________________

c. If it did not work well, state those areas that did not work well:

___________________________________________________________________________

6a. Are you aware of any reproductive health / family planning advocacy and policy

related activity conducted by any NGO/CBO/FBO to your Ministry?

1. Yes 2. No

b. Which of these advocacy contacts have been made to your ministry?

| S/N | Types of advocacy activities | Tick the  appropriate  answer: | Name of / NGO CBO/ FBO | Outcome of the activity |
| --- | --- | --- | --- | --- |
| 1 | Phone calls |  |  |  |
| 2 | Courtesy Visits |  |  |  |
| 3. | Lobbying |  |  |  |
| 4. | Supply of complimentary materials |  |  |  |
| 5. | Campaign |  |  |  |
| 6. | Negotiations |  |  |  |
| 7. | Consensus building |  |  |  |
| 8. | Networking |  |  |  |
| 9. | Others (specify) ____________________ |  |  |  |
| 10. | Others (specify) ____________________ |  |  |  |

c. How many of such advocacy events has direct link with policy making context of

reproductive health / family planning in the state?

______________________________________________________________________

d. What role did you play in linking up such advocacy events to policy making?

7. How would you assess the participating NGOs/CBOs/FBOs in terms of conduct of

advocacy and policy related activities?

8. In your opinion, do you think that the project ““Capacity Building of CSO Partners

to Carry out Reproductive Health /Family Planning Advocacy and Policy Related

activities” has had any impact on improving the skill and capacity of partner

NGOs/CBOs/FBOs (mention those in the state) ?

1. Yes 2. No

(i) If yes, in what ways?

(ii) If No, state reason(s)

___________________________________________________________________________

9. Do you think there were particular challenges that the project posed for your

Ministry?

1. Yes 2. No (If No, go to Ques 10)

(ii) If yes, state the challenges

10. What were the important lessons learned on the project?

11. What is /are the most significant benefit(s) of the project to your Ministry?

___________________________________________________________________________

12(i). Does your Ministry have plan(s) in place for project sustainability?

1. Yes 2. No

(ii). If yes, what plans does the Ministry have to continue with the project or

sustain the tenets of the project?

___________________________________________________________________________

(iii). If No, state reason(s):

___________________________________________________________________________

13. Any other comment / suggestion:

___________________________________________________________________________

## Thank you for your time and input
